# Production, Quality Control and Pharmacokinetic Studies of ^177^Lu-EDTMP for Human Bone Pain Palliation Therapy Trials

**Published:** 2012

**Authors:** Ali Bahrami-Samani, Akbar Anvari, Amir Reza Jalilian, Simindokht Shirvani-Arani, Hassan Yousefnia, Mahmoud Reza Aghamiri, Mohammad Ghannadi-Maragheh

**Affiliations:** a *Radiopharmaceutical Research and Development Lab (RRDL), Nuclear Science and Technology Research Institute (NSTRI), Tehran, Iran. *; b *Department of Radiation Medicine, Engineering Shahid Beheshti University, Tehran, Iran.*

**Keywords:** 177Lu-EDTMP, Radiopharmaceutical, Biodistribution, Pharmacokinetic, Quality control.

## Abstract

Developing new bone pain palliation agents is a mandate in handling end-stage cancer patients around the world. Possibly, Lu-177 ethylenediaminetetramethylene phosphonic acid (^177^Lu-EDTMP) is a therapeutic agent which can be widely used in bone palliation therapy. In this study, ^177^Lu-EDTMP complex was prepared successfully using synthesized EDTMP ligand and ^177^LuCl_3_. Lu-177 chloride was obtained by thermal neutron irradiation (4 × 10^13^ n.cm^-2^s^-1^) of natural Lu_2_O_3_ samples. Radiochemical purity of ^177^Lu-EDTMP was determined by ITLC (more than 99%). Stability studies of the final preparations in the presence of human serum were performed. The biodistribution of ^177^Lu-EDTMP and ^177^LuCl_3_ in wild-type rats was studied by SPECT imaging. A comparative accumulation study for ^177^Lu-EDTMP and ^177^LuCl_3_ was performed for vital organs up to 7 days. The complex was obtained in high radiochemical purity (more than 99%). The complex was stable in vitro in presence of human serum as well as final formulation. Significant bone uptake (> 70%) was observed for the radiopharmaceutical. Due to better physical properties of Lu-177 compared to Sm-153 and acceptable biodistribution results of the compound, ^177^Lu-EDTMP seemed to be an interesting new candidate for clinical trials for bone pain palliation therapy.

## Introduction

Bone metastases are common in the progression of various tumors such as prostate, breast and lung carcinoma. They often entail an occurrence of progressive pain (1) and occur in many patients with solid malignant tumors (2). Approximately 50% of patients with breast carcinoma and 80% of patients with prostate carcinoma develop metastatic bone diseases and nearly half of them experience bone pain (3). Despite the treatment in these patients, a systemic bone avid radiopharmaceutical for the treatment of widespread bone metastases has potential benefits (4). 

Multidentate polyaminopolyphosphonic acid ligands are known to form stable chelates with many metals including lanthanides. Among them, ethylenediaminetetramethylene phosphonic acid (EDTMP) can be envisaged as a possible carrier moiety for the development of beta emitter-based radiopharmaceutical for bone palliation.

Among radioactive lanthanides used in nuclear medicine, lutetium-177 (^177^Lu) is an interesting candidate for therapeutic protocols. ^177^Lu-radiopharmaceuticals have been developed and used in various procedures, such as somatostatin receptor radiotherapy (5), radioimmunotherapy (6), bone palliation therapy (7) and radiosynovectomy (8, 9).

**Figure 1:**
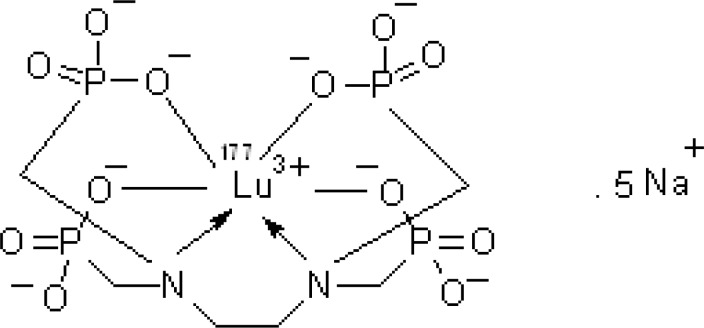
Chemical structure of ^177^Lu-EDTMP

Due to the suitable decay characteristics [t_1/2_ = 6.73 days, E*β *max = 497 keV, E© = 112 keV (6.4%), 208 keV (11%)] as well as the feasibility of large-scale production in adequate specific activity and radionuclidic purity by using a moderate flux reactor, ^177^Lu is a promising radionuclide in the development of ^177^Lu-EDTMP complex as a therapeutic radiopharmaceutical agent (Figure 1).

Compared to Sm-153, Lutetium-177 has a longer half-life which provides the possibility of production of a radiopharmaceutical with better shipment quality and longer shelf-life. On the other hand, irradiation of biological targets by a nuclide with longer half-life may improve the effectiveness of bone pain palliation therapy.

In this research, Lu-177 ethylene-diaminetetramethylene phosphonic acid (^177^Lu-EDTMP) complex was prepared from in-house made starting ligand and the biokinetic studies of the compound were carried out in vital rat organs by post-mortem studies and imaging.

## Experimental

^177^Lu was produced with a specific activity of approximately 70-80 mCi/mg and radionuclidic purity of 99.98% by irradiation of natural Lu_2_O_3_ (99.999% purity from Aldrich Co., UK) targeted at a thermal neutron flux of approximately 4-10^13^ n.cm^-2^.sec^-1^ for 5 days at Tehran Research Reactor (TRR). Whatman No. 3 paper was obtained from Whatman Company (UK). Radio-chromatography was performed by using a Bioscan AR-2000 radio TLC scanner instrument (Bioscan, France). A high purity germanium (HPGe) detector coupled with a Canberra™ (model GC1020-7500SL, Canberra Industries, Inc. CT, U.S.A.) multichannel analyzer and a dose calibrator ISOMED 1010 (Elimpex-Medizintechnik, Austria) was used for counting distributed activity in rat organs. All other chemical reagents were purchased from Merck Company (Germany). 

Calculations were performed based on the 112 keV peak for ^177^Lu. All values were expressed as mean ± standard deviation and the data were compared using Student’s t-test. Animal studies were performed in accordance with the United Kingdom Biological Council’s Guidelines on the Use of Living Animals in Scientific Investigations (1987). The approval of NSTRI Ethical Committee was obtained for conducting this research. 

The wild-type rats (NMRI), weighing 180-200 g, were purchased from Pasteur Institute of Iran (Karaj), and acclimatized at proper rodent diet and 12 h/12 h day/night light/darkness. 

*Production and quality control of* ^177^ *LuCl*_3_ *solution*

^177^Lu was produced by neutron irradiation of 1 mg natural Lu_2_O_3_, according to the reported procedures (10) at TRR (General Atomics, USA). The irradiated target was dissolved in 200 µL of 1.0 molL^-1^M HCl, for the preparation of ^177^LuCl_3_ and then diluted to the appropriate volume with ultra pure water for the production of a stock solution of final volume of 5 mL (0.04 molL^1^). The mixture was filtered through a 0.22 µm filter (Waters, USA) for the purpose of sterilization. The radionuclidic purity of the solution was determined for the presence of other radionuclides using purity germanium (HPGe) spectroscopy, through the detection of various interfering gamma emitting radionuclides. The radiochemical purity of the ^177^LuCl_3_ was evaluated using 2-solvent systems for instant thin layer chromatography (ITLC) [A: 10 mmolL^-1^ diethylenetriamine pentaacetic acid (DTPA), pH = 5 and B: 10% ammonium acetate:methanol mixture (1:1)].

*Synthesis of EDTMP*

EDTMP was synthesized from phosphorous acid, ethylenediamine and formaldehyde in the presence of HCl by a modified Mannich-type reaction (11). To the stirring mixture of phosphorous acid (33.66 g, 0.5 mole) and concentrated HCl (33.44 g) under N_2_ atmosphere, ethylenediamine dihydrochloride (5 g, 0.08 moles) was added drop wise and the mixture was heated to reflux. Aqueous solution of formaldehyde (37 %) was then added drop wise to the mixture. Reflux (at 100 °C) continued for 4 h and the boiling suspension was evaporated under vacuum. The residue was recrystallized from water/methanol mixture [m.p. 214-215°C; IR (KBr, ν cm^-1^): 3308, 2633, 2311, 1668, 1436, 1356; ^1^H-NMR (D_2_O, δ ppm): 3.53 (d, J = 12.3 Hz, 8H,-N-CH_2_-P=O), 3.85 (s, 4H, -N-CH_2_-); ^13^C NMR (D_2_O, δ ppm): 51.63, 52.73; ^31^P NMR (D_2_O, δ ppm): 10.52].

*Radiolabeling of EDTMP with* ^177^ *LuCl*_3_

A stock solution of EDTMP was prepared by dissolving in 1 molL^-1^ NaOH and diluted to the appropriate volume with ultra pure water, in order to produce a solution of 50 mg.mL^-1^. For labeling, an appropriate amount of the ^177^LuCl_3_ solution (0.1 mL, 50 mCi) containing the required activity was added to the desired amount of EDTMP solution (0.3 mL, 1 to 5 mg.mL^-1^). The complex solution was then kept at room temperature for 60 min. The final solution was passed through a 0.22 µm membrane filter and the pH was adjusted to 7-8.5 with 0.05 molL^-1^ phosphate buffer (pH = 5.5). The radiochemical purity was determined using Whatman 3 MM chromatography paper or ITLC-SG, eluted with NH_4_OH (56%): methanol (%100): water (%100) (0.2:2:4; v/v/v) mixture.

*Sterility and Apyrogenicity of the radiopharmaceutical*

Sterility was controlled on a random sampling following the decay of radioactivity. The Limulus Amoebocyte Lysate (LAL) test was used for the validation of radiopharmaceutical production method according to European protocol (12).

*Stability of* ^177^ *Lu-EDTMP in final formulation*

 Stability of ^177^Lu-EDTMP in the final preparation was determined by storing the final solution at 25 °C for 2 days and performing frequent ITLC analysis for the determination of radiochemical purity using Whatman 3 MM chromatography paper or ITLC-SG, eluted with NH_4_OH (56%):methanol (%100):water (%100) (0.2:2:4; v/v/v) mixture.

*Stability of* ^177^ *Lu-EDTMP in presence of human serum*

Final ^177^Lu-EDTMP solution (200 µCi, 50 µL) was incubated in the presence of freshly prepared human serum (300 µL) (Purchased from Iranian Blood Transfusion Organization, Tehran, Iran) at 37 °C for 2 days. The stability was determined by performing frequent ITLC analysis using the above-mentioned chromatography system.

*Biodistribution studies*

The biodistribution of free Lu^3+^ cation as well as of ^177^Lu-EDTMP was determined in wild-type rats. For each radiochemical compound, 100 µL (150 µCi) radioactive solution was injected directly to normal rat through caudal vein. The animals (n = 3) were sacrificed at predetermined times following the injection (2 h to 7 days) and the percentage of the injected dose in the tissues was then determined with a ©-ray scintillation or a dose calibrator.

*Single photon emission computed tomography (SPECT) imaging of* ^177^ *Lu-EDTMP in wild-type rats*

 For imaging studies, ^177^Lu-EDTMP solution (7.4 MBq, 200 µL) was injected intravenously to rats through their tail veins, followed by the injection of propofol-xylazine mixture for anaesthetization. The images were taken after administration of the radiopharmaceutical by a single-head SPECT system (Siemens, Germany) based on 112 keV peak (15% energy window). The rat-to-septa distance was 12 cm. 

## Results and Discussion

**Figure 2:**
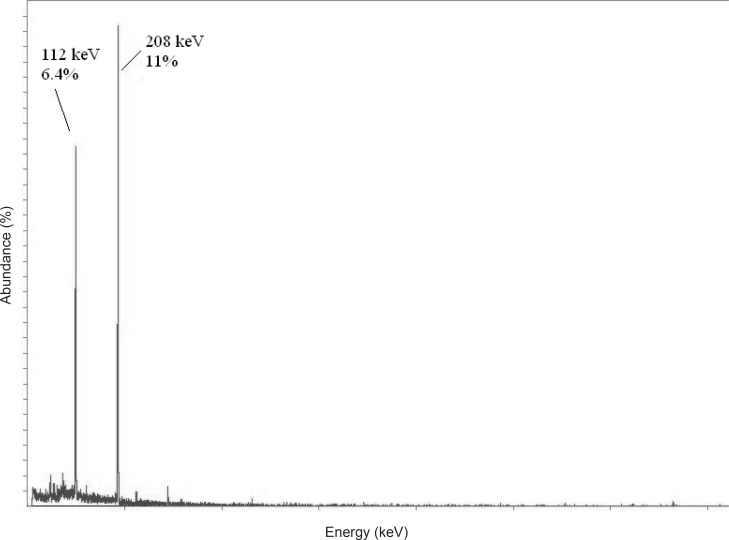
HPGe spectrum for ^177^Lu chloride solution

The radionuclide was prepared at a range of specific activity of 3 to 5 MBq.mg^-1^. For radiolabeling use, after counting the samples on an HPGe detector for 5 h, two major photons (6.4% of 0.112 MeV and 11% of 0.208 MeV) were observed (Figure 2).

**Figure 3:**
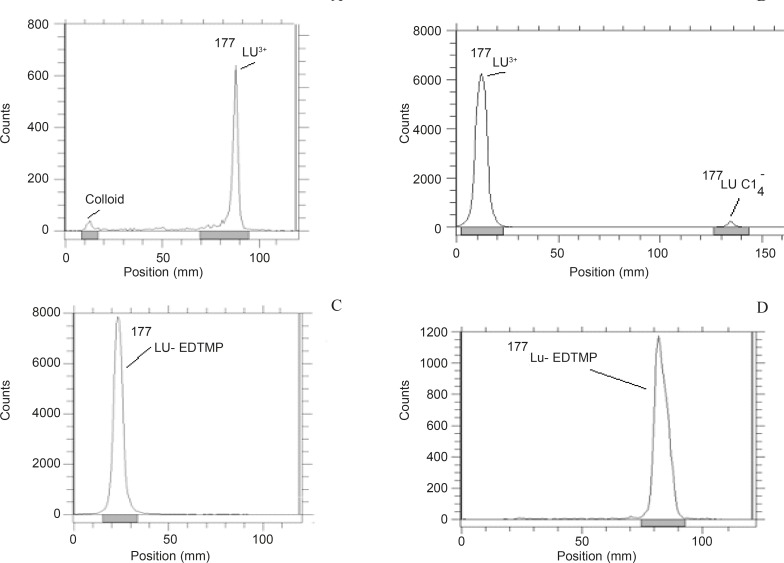
ITLC chromatograms of ^177^LuCl_3_ solution in a DTPA solution (pH = 5). (a) 10% ammonium acetate:methanol (1:1; v/v) solution, (b) chromatograms for ^177^Lu-EDTMP eluted using 10% ammonium acetate:methanol (1:1; v/v) solution, (c) chromatograms for ^177^Lu-EDTMP eluted and with NH_4_OH: methanol:water (0.2:2:4) mixture, (d) chromatography with Whatman 3 MM

The radiochemical purity of the ^177^Lu solution was determined in two solvents. In 10 mmol.L^-1^ DTPA aqueous solution (solvent 1), free Lu^3+^ cation is complexed to more lipophilic Lu-DTPA form and migrates to higher R_f_. Small radioactive fraction remaining at the origin could be related to other Lu ionic species and not due to the forming of Lu-DTPA complex (such as LuCl_4_^-^, *etc*.) and/or colloids. On the other hand, 10% ammonium acetate:methanol mixture (1:1) (solvent 2) was also used for the determination of radiochemical purity. It seems that the fast eluting species was possibly Lu^3+^ and other ionic forms of ^177^Lu (such as LuCl_4_^-^) remained at the origin (R_f_ = 0) as well as colloids (Figure 3). The differences between the impurity peaks in the two chromatograms could be related to the presence of colloidal impurity (2%). Nearly, 2% of activity can also be attributed to other ionic impurities. The effect of various factors on the labeling yield of ^177^Lu-EDTMP including ligand concentration and temperature were also studied and the corresponding results are shown in Table 1.

**Table 1:** Effect of various molar ratios of Lu and EDTMP on the radiochemical yield (30 min at pH = 8-9)

**Lu:EDTMP molar ratio**	**Radiochemical yield (%) **
1:5	98.2
1:10	99.2
1:15	99.7
1:20	99.5
1:40	99.4

As observed, labeling yield increased with increasing molar ratio of Lu:EDTMP (from 1:5 to 1:15) and reached more than 99% in 60 min.

The stability of prepared ^177^Lu-EDTMP complex was studied up to 48 h after preparation. The complex was found to be stable in final pharmaceutical sample and its radiochemical purity was calculated above 99% even 48 hours after the preparation by using Whatman 3 MM eluted with NH_4_OH: methanol: water (0.2:2:4) mixture. Stability test was also developed for the complex in presence of human serum at 37 °C using ITLC as mentioned above.

**Figure 4:**
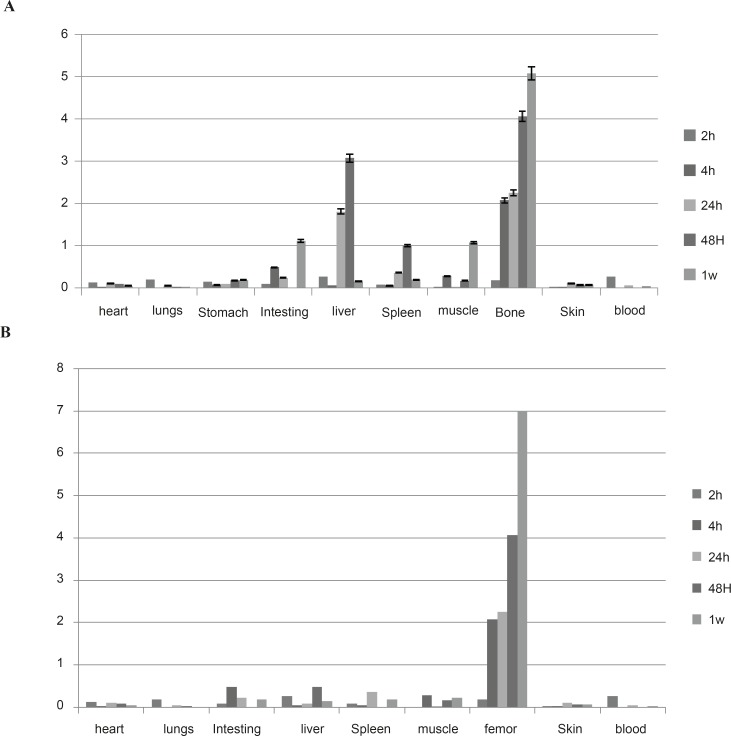
Percentage of injected dose per gram of ^177^LuCl_3_, (a) and ^177^Lu-EDTMP, (b) in wild-type rat tissues after 2, 4, 24, 48 h and 7 days post injection

Regarding the animal studies, the tissue uptakes were calculated as the percent of area under the curve of the related peak per gram of tissue (% ID/g) (Figure 4). 

The biodistribution of ^177^Lu cation was determined in wild-type animals for 2-48 h post injection. The liver radioactivity uptake of the cation is comparable with other radio-lanthanides such as Yb, Sm and Tb (13). About 3% of the cation radioactivity accumulate in the liver in 48 h. 

Low blood radioactivity content demonstrates the rapid removal of ^177^Lu from the circulation after injection. Lung, muscle and skin did not uptake the ^177^Lu significantly, while it is in accordance with other cations accumulation. A 4% bone uptake was observed for ^177^Lu which remained almost constant after 48 h (data not shown). Significant uptake of ^177^Lu uptake was also observed in the spleen, possibly related to reticuloendothelial system. Kidney plays an important role in the excretion of ^177^Lu as a water soluble cation especially after 24 h. The radioactivity biodistribution of ^177^Lu-EDTMP (200 µCi in 150 µL volume) in rat organs up to 7days post injection was determined and it was clearly observed that the major portion of injected radioactivity of ^177^Lu-EDTMP was transferred from blood circulation into bones (Figure 4).

**Figure 5:**
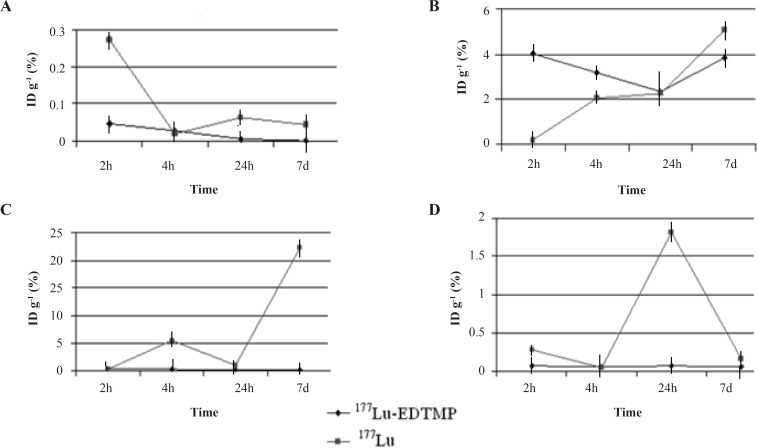
Comparative radioactivity uptake for ^177^Lu-EDTMP and ^177^LuCl_3 _in (a) blood, (b) bone, (c) kidney, (d) liver, and (e) spleen in wild-type rats

Figure 5 compares the ^177^Lu-EDTMP and ^177^LuCl_3_ species behavior in biodistribution and demonstrates the blood accumulation from 2 h to 7 d. Both compounds were washed out from the circulation after 4 h.

^177^Lu-EDTMP was rapidly taken up in bones within 2 h after administration and radioactivity concentration retained almost constant up to 24 h. The accumulation increased slowly after 7 days, whereas the uptake of free ^177^Lu increased slowly. On the other hand, ^177^Lu-EDTMP showed more uptakes at all time intervals compared to free cation, except after 24 h. The error bars at this time point have overlaps, demonstrating the possibility of higher bone uptake even at this stage.

Rapid ^177^Lu-EDTMP uptake and low blood circulation activity lead to a low kidney excretion, whereas ^177^Lu^3+ ^is washed out through kidneys in 7 days as predicted for a cationic species.

Unlike ^177^Lu^3+^ which accumulates significantly in the liver,^ 177^Lu-EDTMP has almost no liver accumulation, which is a major advantage of a therapeutic radiopharmaceutical due to the possibility of increasing the maximum injectable dose.

**Figure 6:**
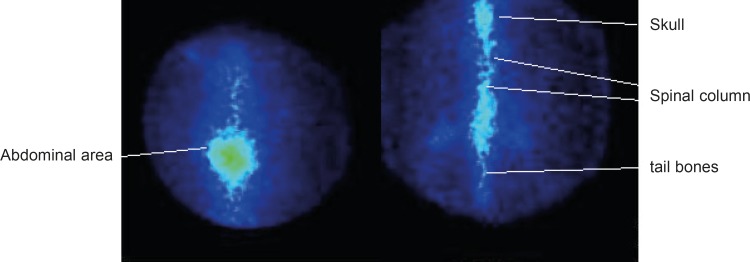
SPECT images of ^177^Lu-EDTMP (right) and ^177^LuCl_3_ (left) 24 h post injection in wild-type rats

As shown in Figure 6, the complex is mainly washed out from the circulation in the first few hours and is trapped in bone tissues especially in spinal column, skull tail and thigh bones. Insignificant activity is accumulated in other tissues. In case of free ^177^Lu, most of activity is accumulated in liver and abdominal area GI.

Recently a similar report on the production and quality control of ^177^Lu-EDTMP complex has been reported (14) demonstrating higher localization of ^177^Lu-EDTMP in skeleton compared to ^153^Sm-EDTMP which is also in agreement with our data. 

In a recent study on the species-specific pharmacokinetic behavior of the drug in canine model did not demonstrate any biological adverse effects suggesting ^177^Lu-EDTMP as a promising radiopharmaceutical for further human studies (15).

As a comparison, the labeling and QC of the ^177^Lu-EDTMP complex was more or less similar to the Sm-153 analog and no significant difference was observed in the biodistribution data. The radiopharmaceutical higher bone uptake and half life may impose long term bone marrow and other critical organs irradiation. Thus, theoretical and practical dosimetry studies should be considered for this ligand at various injected doses to humans.

## Conclusions

EDTMP ligand was synthesized and its structure was determined using authentic spectroscopic methods. ^177^Lu-EDTMP was prepared (radiochemical purity more than 99%) using optimization studies. ^177^Lu-EDTMP and ^177^LuCl_3_ preparations were administered intravenously through the tail vein to wild-type rats and biodistribution data collected after 2 h to 7 days showed at least 70% accumulation of the drug in the bone tissues. SPECT images were taken from wild-type rats injected with ^177^Lu-EDTMP and ^177^LuCl_3_ after 24 h and the biodistribution was shown to be consistent with *post-mortem* data. A comparative accumulation study for ^177^Lu-EDTMP and ^177^Lu was performed for vital organs up to 7 days. ^ 177^Lu-EDTMP seemed a promising agent for bone pain palliation therapy in skeletal metastases in humans. Thus the radiopharmaceutical was released for clinical trial process in 20 volunteer human with metastatic bone malignancies in Shiraz, a central city in Iran (16).
